# Promoting Creativity Through Transcranial Direct Current Stimulation (tDCS). A Critical Review

**DOI:** 10.3389/fnbeh.2018.00167

**Published:** 2018-08-02

**Authors:** Claudio Lucchiari, Paola Maria Sala, Maria Elide Vanutelli

**Affiliations:** Department of Philosophy, Università degli Studi di Milano, Milan, Italy

**Keywords:** creativity, divergent thinking, convergent thinking, brain stimulation, tDCS, DMN

## Abstract

Creativity, meant as the ability to produce novel, original and suitable ideas, has received increased attention by research in the last years, especially from neuroaesthetics and social neuroscience. Besides the research conducted on the neural correlates of such capacities, previous work tried to answer the question of whether it is possible to enhance creativity through cognitive and neural stimulation. In particular, transcranial direct current stimulation (tDCS) has been applied to increase neuronal excitability in those areas related to creativity. However, being a complex construct that applies to a huge variety of situations, available results are often confusing and inconsistent. Thus, in the present critical review, after selecting original research articles investigating creativity with tDCS, results will be reviewed and framed according to the different effects of tDCS and its underlying mechanisms, which can be defined as follows: the promotion of self-focused attention; the disruption of inhibiting mechanisms; the enhancement of creative thinking; the promotion of artistic enactment. Finally, a theoretical perspective, the creative on/off model, will be provided to integrate the reported evidence with respect to both anatomical and functional issues and propose a cognitive explanation of the emergence of creative thinking.

## Creativity: A Theoretical Framework

Creativity has been defined by some scholars as humankind’s ultimate resource (Toynbee, 1964). It is traditionally considered a mere outcome of an individual’s genius, a gift, and a personality trait. Consequently, the interpretation that emerged in relation to this topic tended to consider creativity as something given without a reason, even genetically predetermined, and thus far from objective and systematic comprehension (Batey and Furnham, 2006). However, such ideas deal only with the first of the four points that Rhodes (1987) suggested for a complete framing of creativity. According to his proposal, it is possible to conceptualize creativity on the importance given to: (a) the person who creates; (b) the cognitive processes involved in the creation of ideas; (c) the environment in which creativity occurs; and (d) the outcome of the creative activity. Besides the specific focus on these different contributions to the phenomenon, it is also important to consider the multifaceted expressions of creativity, from artistic enactment, to scientific progress, and to problem-solving. Therefore, although there is a great interest dedicated to this issue, it is still difficult to define and study creativity in a comprehensive way. The most accepted definition that can be easily applied to all contexts proposes a double requirement for creativity: originality and usefulness (Barron, 1955). Moreover, different perspectives proposed creativity as being the result of a complex interaction between people and environment (Csikszentmihalyi, 1988; Mumford and Gustafson, 1988), revealing the importance of cognitive, social and cultural factors in creative studies (Amabile, 1983, 1996).

**Figure 1:**
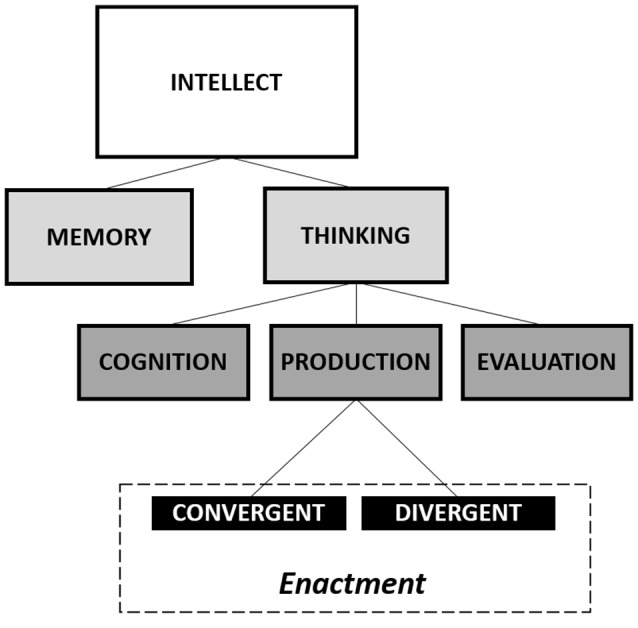
Guilford’s diagram of the intellectual factors. Modified from Guilford (1957).

In the present article, we meant to emphasize the dynamic nature of creativity and the possibility of enhancing it. Starting by the Guilford (1957) model, which collocates the creative thinking within the interaction of specific cognitive processes (see Figure 1), we may describe the cognitive system as a substantially stable arrangement of components involved in many purposed tasks, which may be disrupted by a perturbation that produces a destabilization of the whole system, resulting in creative functioning.

For example, a problem that cannot be solved using the already available cognitive algorithms may produce an attention shift toward inner processes, the search for alternative solutions, and the subsequent activation of new neurocognitive paths. The same effect may be observed in artistic creativity. In this case, it is not important to reach a definitive solution, but there is anyhow an implicit need to express an indistinct thought in a synthetic form. In fact, we propose the presence of a specific need-for-enactment, which aims at associating a widespread cognitive network with a motor program that can provide a behavioral output.

The aim of the present study is to review the most eminent work on the effects of transcranial direct current stimulation (tDCS) on creativity. Here, the neurocognitive underpinnings and empirical research are considered with special interest, since we aimed at demonstrating that it is possible to target this complexity by using tDCS. This noninvasive brain stimulation technique can modulate cortical excitability by influencing neuronal membrane potential within different neural networks. Anodal stimulation can increase excitability and result in a depolarization, while cathodal stimulation decreases it and leads to a hyperpolarization of the resting membrane potential (Sehm et al., 2012). Interestingly, previous research highlighted that anodal tDCS is associated with locally reduced GABA neurotransmitter (Stagg et al., 2009; Kim et al., 2014), while cathodal stimulation causes reduced glutamatergic neuronal activity (Stagg et al., 2009). In this view, brain modulation may be extremely useful both in understanding the functional neurophysiology of creativity and in developing tailored programs aimed to elicit a targeted creative experience. Since creativity cannot be captured by a single concept, being multifaceted and complex in nature, it could be considered as a meta-construct made by an aggregate of processes. Thus, we need a process-driven approach (Dietrich, 2004) to test if and how we can enhance it by tDCS. More specifically, we argue that brain modulation may be effective in: (1) promoting self-focused attention; (2) decreasing the effect of perceptual filters and inhibiting mechanisms; (3) enhancing creative thinking by facilitating the emergence of imagination and analogical mechanisms; and (4) favoring the way toward the enactment. Following this schema, we have grouped the available work and framed it accordingly.

## Method

This critical review is composed of two main sections: the first one describes evidence related to each of the key processes implied in creativity studies by describing the main published articles in the area. In the second part, we will provide a general model that integrates the evidence discussed thus proposing an explanation of the effect of tDCS on creativity. Searches were conducted through PubMed, SCOPUS, PsycINFO and Google Scholar. The following keywords or combinations were used: neuromodulation, neurostimulation, tDCS, creativity, divergent thinking and artistic expression. We considered articles without any time limits, excluding previous reviews. Articles in languages other than English, letters and editorials were excluded. We considered as eligible for inclusion all original research articles reporting data about the use of tDCS to study any creativity-related cognitive process. Both studies on healthy and pathological subjects were considered. The first search identified 48 articles. An initial review of the abstracts of these articles by authors identified 22 articles that were potentially relevant to the current review. The abstracts of these articles were then evaluated by authors, resulting in 14 articles being identified as eligible for inclusion, with an additional four articles identified during manuscript preparation, for a total of 18 articles. This review provides a synthesis of the findings from previous key reviews and empirical studies identified in the literature search (see Table 1).

**Table 1:** Overview of original creativity research conducted by transcranial direct current stimulation (tDCS) technique.

Cognitive process	Cognitive function	Task	Task duration	tDCS paradigm	tDCS duration	tDCS intensity	Brain area	Sample size
**Promoting self-focused attention**								
Kajimura et al. (2016)	Mind Wandering	Mind-Wandering Measurement Task (MWMT)	20 min	offline	20 min	1.5 mA	IPL	*N* = 60; 3 groups
Axelrod et al. (2015)	Mind Wandering	Sustained Attention to Response Task (SART)	40 min	online	20 min	1 mA	DLPFC	**Exp1**: *N* = 14 **Exp2**: *N* = 31
Bertossi et al. (2017)	Mind Wandering	Choice reaction time task (CRT)	25 min	offline	15 min	2 mA	mPFC	*N* = 72; 3 groups
**Decreasing inhibiting mechanisms**								
Mayseless and Shamay-Tsoory (2015)	Verbal divergent thinking	Verbal Fluency Task Alternate Uses Task (AUT) Visual figural task (VFT)	Verbal fluency task: 2 min AUT: 4 min VFT: 10 min	online	22 min	1.5 mA	IFG	**Exp1**: *N* = 30; 2 groups **Exp2**: *N* = 30; 2 groups
Ivancovsky et al. (2018)	Verbal divergent thinking	AUT	20 min	online	20 min	1.5 mA	IFG	*N* = 60; 2 groups
Chrysikou et al. (2013)	Cognitive Flessibility	Uses Task	12 min	online	20 min	1.5 mA	PFC	*N* = 48; 6 groups
**Enhancing creative thinking**								
Colombo et al. (2015)	Divergent thinking	AUT	-	offline	20 min	1.5 mA	DLPFC	*N* = 45; 3 groups
Zmigrod et al. (2015)	Divergent/convergent thinking	Compound Remote Associative Task (CRA)	CRA: 5 min AUT: 4 min	online	20 min	2 mA	DLPFC PPC	*N* = 28
Cerruti and Schlaug (2009)	Verbal Associative Thought	The remote associates test (RAT) Verbal Fluency Task (VF)	VF: 3 min RAT: 8 min	offline + online	20 min	1 mA	DLPFC	**Exp1**: *N* = 18 **Exp2**: *N* = 12
Green et al. (2017)	Creative Analogical Reasoning State Creativity	Analogy Finding Task Thin Slice Creativity Verb Generation Task	AFT: 5 min Verb: 16 min	offline + online	20 min	2 mA	Frontopolar Cortex	*N* = 31; 2 groups
Ruggiero et al. (2018)	Convergent and Divergent Thinking	Divergent Thinking Test (DTT) Remote Association Test (RAT)	DTT: 20 min RAT: no time limit	offline + online	20 min	1.5 mA	ATL	*N* = 21; 3 groups
Chi and Snyder (2011)	Insight	Matchstick arithmetic	-	offline+online	17 min	1.6mA	anterior temporal lobe (ATL)	*N* = 60; 3 groups
Aihara et al. (2017)	Verbal and Non-Verbal Insight	Matchstick arithmetic RAT Two-digits addiction arithmetic task	MA: 8 min RAT: 5 min Addition: 2 min	offline+online	25 min	1.6mA	ATL	*N* = 66
Goel et al. (2015)	Insight Divergent Thinking	Riddles/Insight Task Phonemic Verbal Fluency Task	Riddles: 37 min Fluency: -	offline	20 min	2 mA	lMTG rTPJ	*N* = 32; 2 groups
Zmigrod et al. (2015)	Convergent and Divergent Thinking	Compound Remote Associative Task (CRA) Alternative Uses Task (AUT)	CRA: 5 min AUT: 2 min	online	20 min	2 mA	DLPFC (Exp 1) PPC (Exp 2)	**Exp1**: *N* = 14 **Exp2**: *N* = 14
Ghanavati et al. (2017)	Figural Fluency	Five Point Test (FPT)	FPT: 5 min	online	15 min	1.5 mA	r-PPC l-DLPFC	*N* = 20
**Promoting artistic enactment**								
Rosen et al. (2016)	Creativity	Compound Remote Associative Task (CRA) Verb Generation Task Stroop Test	Total time: 25 min	offline	15 min	1.5 mA	r-DLPFC	*N* = 17
Anic et al. (2017)	Creativity and Technical Fluency	Musical Improvisation	Total experiment time: 90 min	online	15–20 min	1.4 mA	M1	*N* = 8; 2 groups

## Neuromodulation to Promote Self-Focused Attention

In this paragraph, we focus on the ability of subjects to detach attention from external stimuli. Indeed, creative thinking often requires individuals to focus their attention on cognition, shifting from the external environment to the internal imaginative processes. We start from the idea that this task needs a mechanism able to support and actively maintain it, thus allowing the brain to activate creative processes while still remaining in touch with the environment. Here, we believe the default mode network (DMN) might play a fundamental role. The DMN comprises hubs and subsystems in interplay, which have been related to “internal mentation” (Andrews-Hanna, 2012). The interaction between creativity and the DNM refers to spontaneous imagination and self-generated thought (Andrews-Hanna, 2012; Andrews-Hanna et al., 2014). The interaction between creativity and the DNM refers to spontaneous imagination and self-generated thought (Andrews-Hanna, 2012; Andrews-Hanna et al., 2014). Interestingly, different regions of the DMN were found to be associated with creativity. For instance, divergent thinking is associated with a resting-state functional connectivity (RSFC) between the medial prefrontal cortex (mPFC) and the posterior cingulate cortex (PCC; Takeuchi et al., 2012). Similarly, Beaty et al. (2014) found greater RSFC between the left inferior frontal gyrus (IFG) and the entire default mode network in a high creativity group. Furthermore, research also suggests that increased RSFC between the mPFC and the middle temporal gyrus (mTG) might be crucial to creativity, defining a potential target for brain modulation with the aim of promoting creativity (Wei et al., 2014). Also, it seems that the topological properties of brain networks are highly associated with creative ability. For example, the small-world organization of the DMN allows information integration and segregation, thereby addressing the work to subnetworks within the global cognitive system. This result is a particularly desirable one when facing standard situations. However, it limits the increase in creative solutions. Indeed, it has been shown that high-creativity subjects have shorter links within local nodes and stronger connections with distal functional networks, which lead to a facilitated spread of information within the whole system (Hermundstad et al., 2012). This activity diffusion supports a flexible generation of original ideas, a task that requires the wide distribution of attentional resources (Jung et al., 2013), and the retrieval of potentially useful information (Andrews-Hanna et al., 2014). Here, the DMN proved to play a key role in producing interindividual variation during the creative performance. Finally, interesting associations have been found between personality traits, DMN functioning, and creativity. For example, a study by Beaty et al. (2014) highlighted that the openness personality trait, which is positively linked to creative performance, is related to the global efficiency of the DMN.

In this framework, we may hypothesize that tDCS training could be effective in promoting and enhancing DMN flexibility. In particular, research by Kajimura et al. (2016) explored the relationship between the activity of some areas belonging to the DMN—more specifically, the right inferior parietal lobe (rIPL)—and mind-wandering. They applied anodal tDCS over the right rIPL and cathode over the left lateral prefrontal cortex (lLPFC) as well as the opposite montage. Behavioral results showed reduced mind-wandering after applying the first montage when compared to the opposite one, while at the neural level some decreased functional connections from the right IPL were found. In fact, as discussed by the authors, it seems that the decrease in mPFC-PCC connectivity mediated the decrease in mind wandering, thus suggesting a facilitation from their afferent connections to this mind functioning. These results may suggest that the modulation of specific regions of DMN may actually change the whole system balance, changing the attention focus and thus promoting or inhibiting imaginative processes.

Another interesting relationship is the one between the DMN and the executive functions modulated by the dorsolateral prefrontal cortex (DLPFC). Axelrod et al. (2015) applied the anode electrode to the left DLPFC and the cathode electrode to the right supraorbital area and found that the depolarization of the left DLPFC increased mind wondering propensity during a sustained attention task. During this task, subjects responded to relatively rare items within a long and boring list of repetitive trials. This situation is known to produce task-unrelated thoughts. In their study, authors found that the propensity to produce unrelated thoughts was increased during anodal tDCS of the left DLPFC without impairing attentional performance. Authors suggested that this effect might be linked to an indirect effect of the stimulation on the default mode network. Furthermore, since the mind-wandering evoked by tDCS did not impair the task performance, it is possible that the specific montage enhanced the cognitive resources of subjects, allowing both proper responses and unrelated thoughts. Generally speaking, the stimulation of prefrontal regions may influence the balance of the default network and modulate executive functions at the same time, an effect that might be desirable in creative tasks. Another recent study performed on a mind-wandering task (Bertossi et al., 2017) also highlighted the crucial role of the mPFC, since the administration of cathodal tDCS decreased the attitude to mind-wander. Moreover, the effect was also accompanied by a change in the content perspective, which became more related to other people rather than self-related issues. The idea that tDCS may produce an attention modulation to favor creative processes seems then to be supported by evidence.

## Decreasing the Effect of Filters and Inhibiting Mechanisms

Another possible way to promote creative answers is by disrupting the inhibiting mechanisms that try to maintain individual thinking inside-the-box. In this way, the usual cognitive algorithm may be efficiently applied to incoming stimuli, inhibiting the search for further algorithms and filtering out less salient information. The left IFG (lIFG) and the anterior insula were reported to play key roles in these mechanisms (Abraham et al., 2012; Uddin, 2015). Since the insula may not be directly modulated by tDCS, studies targeted the IFG.

For example, a study by Mayseless and Shamay-Tsoory (2015) stimulated the lIFG to test the effect of tDCS on a divergent task. Authors moved from the neurofunctional assumption that creativity relies on a balance between right and left hemispheric activation (The Balance Hypothesis). Thus, the aim of their study was to test this model by altering this balance through tDCS at the lIFG during a divergent thinking performance. Researchers applied a bilateral stimulation with the cathode over the right IFG, and the anode over the left, and compared this condition with the opposite one. Results showed increased divergent scorings when the left IFG was deactivated by the cathodal stimulation, while the opposite condition did not affect the creative performance. To better explore these issues, in a second experiment authors administered unilateral stimulation with either the anode or the cathode over the left and right IFG alone. The stimulation of each area alone was not sufficient to modify the creative process Results supported the Balance Hypothesis since neither of the two conditions resulted in an enhanced or impaired creative performance.

Another study by Ivancovsky et al. (2018) targeted the same area (the lIFG) during the alternate uses task (AUT; Guilford et al., 1978). In this task, common objects are shown and participants are asked to report as many alternate uses as possible within a 4-min period. In their study, researchers applied a 20-min cathodal stimulation to the lIFG with the anode at the right supraorbital region as well as the opposite montage. Results showed an increase in creativity when the left IFG was hyperpolarized, while the depolarization of the same area decreased creativity scores. Considering that the lIFG was previously associated with inhibitory processes (Aron et al., 2003; Swick et al., 2008), we may suggest that the application of tDCS over the lIFG produces a lowered inhibition mechanism, thus allowing more flexible and creative thinking.

The notion that cognition may be modulated by disrupting top-down cognitive control, allowing the brain to process unfiltered bottom-up information, is also supported by a number of studies targeting the prefrontal cortices. For instance, Leite et al. (2011) found that the anodal stimulation of the DLPFC coupled with the cathodal stimulation of the primary motor area increases the efficiency in task shifting. Similarly, Chrysikou et al. (2013) measured the performance results of the uncommon use (UU) task, contrasted with the common use (CU) task, during the cathodal stimulation of the left or the right prefrontal cortex (F7/F8), with the cathode on the contralateral mastoid. They obtained that the hyperpolarization of the left prefrontal cortex led to an increase in the number of the UUs reported as well as in the speed of responses. Interestingly, the number of common uses reported was not affected by tDCS. The study supports the idea that the disruption of the prefrontal control system facilitates cognitive flexibility.

## Enhancing Creative Thinking

A third fundamental strategy to affect creativity through neuromodulation is to directly target thinking processes. Traditionally, creativity has been associated with divergent thinking, which is the ability to follow unconventional ways of reasoning, thus producing new ideas and solutions. It is obvious that creativity also implies convergent thinking, i.e., the ability to select ideas for their appropriateness in relation to a given task (Finke et al., 1992; Amabile, 1996; Sternberg, 1999). To affect thinking processes different strategies are possible, from modulating attention to favor semantic connections and information integration. For the sake of clarity, in this section we will provide evidence categorized by the targeted cortical area.

### Prefrontal Areas

As discussed above, neuromodulation may be effective in promoting both ideas generation (by enhancing analogical associations), and idea selection. Since these processes require high order information processing, studies often targeted the DLPFC. Colombo et al. (2015) investigated the role of the DLPFC in a task that required subjects to find original uses for common objects (so-called AU task). Twenty minutes of anodal tDCS over the left DLPFC increased creative responses, but only when the task was primed by a condition able to promote divergent thinking. This means that tDCS stimulation had an effect only when interacting with a creative primer. These results may be related to the role of the DLPFC in coordinating attentional shifts since attentional focusing was associated with a worsened performance of the AU task in previous studies (Friedman et al., 2003). However, it is likely that when the cognitive system is called to work on a similar task, the DLPFC also plays a role at a higher level. To use a metaphor, the DLPFC might be responsible for modulating a kind of cognitive search engine aimed at associating an object with possible uses. Normally, the first reference that comes to mind is related to the frequency of use and/or prototypical features of the probe, something like the sponsored links displayed by Web search engines. Sure, we can be aware of many other links (e.g., uses), but generally, we are “forced” to stop searching the list after having read the top of it, leaving uncommon and probably more creative uses out of mind. However, when the DLPFC is impaired, the search engine is free to search deeper and propose a randomly ordered list for frequency and/or prototypical features, thus allowing creative cognitive processes. The DLPFC might then be linked to the maintaining of a given view of the world by searching for a confirmation of previous expectations more than trying new, original cognitive solutions. It would implement a sort of cognitive inhibition on a large-scale. Actually, a study by Zmigrod et al. (2015) reported increased performances on the Compound Remote Association task (CRA) when anodal tDCS stimulation was applied at the left DLPFC with the cathode at the right DLPFC. Since the CRA is intended to measure convergent thinking (asking responders to find a link between three unrelated words), authors suggest that the DLPFC plays a key role in analytical information processing. Similar results were found in a previous study by Cerruti and Schlaug (2009) who used a unilateral montage over the lDLPFC to modulate the performance of the Remote Association Task. Consequently, the role of the DLPFC appears to be complex and linked to highly demanding tasks, e.g., the integration of semantically distant information, creative idea selection, and convergent thinking. All of these processes require the brain to search in depth for higher-level connections.

### Temporal Areas

A study by Chi and Snyder (2011) reported increased problem-solving abilities by insight in subjects who received cathodal stimulation over the left anterior temporal lobe (ATL) together with anodal stimulation of the contralateral region. The authors refer this increase in performance to a general modulator role of the left ATL, which is involved in a top-down inhibiting system that facilitates the use of routinized solutions. Authors suggest that the contemporary stimulation of the left ATL and DLPFC could induce even greater and/or generalized effects on creativity. The basic idea is that many neural circuits compete during a cognitive task. The increase of one over the others depends on both experience and anatomical (genetically driven) features. Thus, people with a middle to strong left dominance (in their study Chi and Snyder, 2011 controlled this variable by selecting only right-handed people with 50 or more on the Edinburgh Scale) generally show less hypothesis switching than less lateralized people due to the strong role played by the left ATL and frontal areas in top-down inhibiting processes. These mechanisms are tuned by experience and generally allow people to obtain good (or even excellent) results in their routinized activities. Actually, in the same study, people who received left cathodal and right anodal stimulation did not worsen their performance, since the left ATL seemed to be already optimized. Thus, a one-shot stimulation (20 min of tDCS) cannot obtain a substantial change in performance. In less lateralized people, it is possible that a lower top-down modulation allows for easier perspective shifting, thus facilitating insight solutions and ideas generation. At the opposite, an excessive imbalance toward right ATL functioning might impede or hinder the acquisition and/or the tuning of cognitive algorithms useful in repetitive daily or professional situations. However, a study by Aihara et al. (2017) on a sample of 66 Japanese subjects did not find any effect on insight after tDCS applied over ATL. In fact, neither the right ATL anodal/left ATL cathode nor the reversed montage affected the subjects’ performance at the remote associates test (RAT; verbal insight) and at the matchstick task (nonverbal insight). Authors suggested that their results might be due to the specific experimental conditions adopted. This underlines the fundamental role of the experimental setting when testing the efficacy of tDCS programs. Actually, another study (Ruggiero et al., 2018) targeted the same areas obtained different results. Authors used a verbal task (the RAT) and a graphical task (the Divergent Thinking Test, DTT) and they found a positive effect of the anodal stimulation on the ATL with the cathode on the right ATL at RAT performance. However, results showed that this effect was significant only for reducing the time needed by subjects to produce a verbal insight (RAT), while accuracy was not affected. Furthermore, the DTT performances were not affected by tDCS, supporting the idea that the left ATL is involved in verbal creativity. Taken together, such evidence suggests that suppressing the left anterior temporal region could disengage the correspondent right region from the control of the left inhibiting system that normally limits access to cognitive resources, thus improving creative thinking skills. The left frontotemporal region was also shown to be linked to creative analogical reasoning and information integration since its activation correlates with semantic distance in an analogy generation task (Green et al., 2010, 2015). Finally, Goel et al. (2015) proposed different creative tasks in both divergent and convergent thinking domains to English speakers and 16 non-native English speakers. They wanted to test the effect of a neuromodulation protocol on the ability to solve linguistic riddles. The tDCS was administered over the left mTG and the right temporoparietal junction (TPJ). The stimulation was able to modulate the performance of the task in specific ways: while performing the insight task (convergent thinking), the stimulation of the right TPJ and the deactivation of the MTG improved results; conversely, during the divergent thinking task, the same stimulation resulted in decreased performances. Authors explained their results, considering the role of the MTG, which is involved in routine semantic processing. Instead, the TPJ is associated with the search for unusual and/or metaphorical meanings of the language. Consequently, the hyperpolarization of the left MTG may have facilitated a shift in perspective, allowing for the activation of an active search for alternative meanings. This effect may be particularly acute in non-native speakers since they lack the knowledge of idiomatic expressions and their poor language expertise does not easily allow them to go beyond the literal meaning. Favoring the work of the right TPJ and partially inhibiting the top-down supervision of the left MGT, non-English speakers gained a freer, creative interpretation of phrases.

### Parietal Areas

The study by Zmigrod et al. (2015) investigated the influence of tDCS on performances in verbal tasks targeting the posterior parietal cortex (PPC). Authors found that the anodal stimulation of both the left and right PPC increased insight and decreased analytical answers when compared to the control condition. Considering that the PPC is related to goal-directed attentional processes (Behrmann et al., 2004), it could be hypothesized that the depolarization of this region might increase the ability of the person to disengage bottom-up automatic mechanisms. This disengagement may favor divergent thinking and insight solutions. Ghanavati et al. (2017) applied tDCS over the same brain site (both right and left PPC) during a figural fluency task. Results showed that participants produced more unique designs under anodal rPPC tDCS. Such findings support the idea that the PPC plays an important role in both verbal and visual creativity by modulating attention mechanisms.

## Promoting Artistic Enactment

The last important issue related to creativity deals with enactment, which is the ability to finalize the creative course in a tangible and concrete way. Enactment may relate to any sphere of life, but it is particularly interesting when referred to artistic production since it merges different cognitive and motor processes in a unique and appreciable shape. At this regard, a case study provided original suggestions: Simis et al. (2014) from the Harvard Medical School had the opportunity to study a man affected by a stroke of the left middle cerebral artery. After the stroke, this person, without any art education, reported the urge to draw and started training and perfecting his drawing and painting skills using his left hand (previously he was right-handed, but the right hand was impaired). Four years after the stroke, the patient underwent a tDCS protocol consisting of the anodal stimulation of the right (unaffected) frontotemporal region. Here, we have the effect of the stroke over the left hemisphere and the neuromodulation of the right frontotemporal region. The former produced the urge to draw and express the patient’s new perspective of the world. The neuromodulation increased his drawing abilities and creativity. In particular, 30 min of anodal stimulation produced increased graphical abilities in a drawing task. Though it is difficult to provide a complete explanation of this effect based only on the interplay between the two hemispheres, it is possible that the impairment of the left frontotemporal region may disinhibit artistic motivation, which might partially substitute the communication functions affected by the stroke. At the same time, the anodal stimulation of the right frontotemporal area probably enhanced the function of the frontoparietal connectivity, which is supposed to play a fundamental role in perspective elaboration and is, consequently, vital in graphical representation. Results revealed in this work are in line with recent evidence acquired from the application of tDCS for improving behavioral performance and functional connectivity after stroke in both motor (Chen and Schlaug, 2016) and cognitive domains (Marangolo et al., 2016). For example, a recent work by Marangolo et al. (2016) about the effects of tDCS on language recovery and resting state functional connectivity found a positive effect of real vs. sham stimulation on behavioral performance, as well as on the interconnectedness of several regions of motor, default and control network.

Moving to music enactment, Rosen et al. (2016) proposed a dual process model for creative production in jazz improvisers: Type-1 would be based on automatic, associative processes, while Type-2 on executive, controlled ones. The use of either Type 1 or 2 is thought to be associated with domain expertise: novices would rely more on top-down, controlled mechanisms, while expert musicians to bottom-up, implicit processing. To test their hypothesis, authors performed a tDCS protocol by targeting the right DLPFC with anodal, cathodal, or sham stimulation. Results showed a significant interaction between the musicians’ expertise and tDCS: after anodal stimulation, novices performed better in terms of originality as assessed by expert musician judges, while the opposite effect was obtained for the most experienced musicians, who reported poorer performance. Researchers interpreted their findings as a confirmation of the dual model since different modes of creative expertise were found between experts and novices. Also, it was proven that anodal stimulation may effectively modulate rDLPFC activity, which is recruited during improvisation. Remaining within the theme of musical improvisation, Anic et al. (2017) explored the role of another brain area, the Motor Cortex (M1), for the creative process. In particular, starting from the idea that it subserves the acquisition and consolidation of novel movements, they applied a bilateral tDCS montage (left and right M1) to two different groups of expert pianists while requiring them to improvise some musical sequences. The left and right M1 areas received either anodal or cathodal stimulation according to the group (anodal-left/cathodal-right; cathodal-left/anodal-right). Although this was a pilot study (only eight musicians took part in the experiment, four for each group), preliminary analyses showed that left excitatory stimulation increased creativity and technical fluency while cathodal stimulation apparently didn’t produce any significant behavioral modifications. Accordingly, authors concluded that there is at least preliminary evidence that the M1 region contributes to musical fluency and creativity. However, due to the sample size, future research should better explore these issues to identify significant statistical effects. Being a primary motor region M1 is, without any doubt, related to an executive component of musical creativity. Thus, the excitatory processes within this area could also, in this case, lead to enactment. Considering the underlying neurophysiological mechanisms of such processes, previous research (Nitsche et al., 2005) underlined that the corticospinal excitability modulation provided by tDCS over M1 can be attributable to membrane polarization, and less on synaptic modifications. On the other side, the engagement of intracortical synaptic mechanisms has been found for tDCS after-effects.

## Conclusion: Switching on the Creativity State

Starting from the evidence discussed above, we conclude this review with a model aimed at explaining how tDCS, as well as other neuromodulation techniques, may favor creativity. We begin with the consideration that creativity is influenced by both stable characteristics and contextual conditions. Regarding stable traits, (Aron and Aron, 1997) suggested that people could differ in the way they neurologically transmit and process sensory information (Gerstenberg, 2012). It is the case of sensory processing sensitivity (SPS), described as a personality trait modulated by genetic factors, which allows people to feel and process more information at one time, and in a deeper way. Such sensitivity is referred to both external and internal stimuli (Jagiellowicz et al., 2011). Accordingly, Highly Sensitive Persons (Aron and Aron, 1997) are more inclined to experience higher arousal during exposure to environmental stimuli such as bright lights, strong smells, noise and chaotic situations. Moreover, they startle easily and are strongly sensitive to caffeine and time pressures (Jagiellowicz et al., 2011). Finally, those with SPS are more reactive to interpersonal and emotional cues. For example, they are more susceptible to the presence of external observers when performing tasks, and more aware of others’ moods. These peculiar reactions are already present at the very first stages of life (Aron et al., 2012; Davidson et al., 2002) and are associated with specific neurobiological markers (Herberner et al., 1989; Aron et al., 2010). Such sensitivity could sometimes result in a sensorial overload with subsequent negative effects, like exertion and fatigue. Thus, it is possible that they could more often feel the urge to rest alone and sleep longer throughout the day (Aron and Aron, 1997). The existence of these differences is noteworthy since a tDCS program aimed to improve creativity may have different effects on different individuals. Not checking for these differences might also lead to different study results.

However, our idea is that creativity, though influenced by stable individual characteristics, may be seen as a transient property of the cognitive system, a mental configuration we may call a “creativity-on” state, which may be spontaneously activated and/or evoked by external conditions (e.g., by a task that asks individuals to find creative semantic connections between words). So far, people may be trained to have easier access to this state when they need it. We may define a state of creative-on, as opposed to a state of creative-off. The latter is essentially based on ordinary functioning, e.g., the search for information in long-term memory or the use of prompt cognitive algorithms. From a neurofunctional point of view, the creative-on/off dynamic is substantially based on the interaction between the DMN and the cognitive control system (Beaty et al., 2014, 2016). The core structures of DMN are the mPFC, the PCC precuneus (prec), and the posterior inferior parietal lobule (pIBL). The role of the network is multifaceted since it is involved in a number of specific cognitive processes. However, different studies showed that an increase of DMN activity, in particular, the connection strength between mPFC and PCC/pre, is involved in creative processes (Beaty et al., 2015). Conversely, the cognitive control system involves the left IFG and its role is to inhibit cognitive processes not linked to the ongoing cognitive plan. In particular, the modulatory role of the IFG is active in the ATL, in particular, the temporal pole (TP), which is considered a sort of semantic hub. When the left IFG system activity is high, the semantic search and integration will involve only short-range semantic connections (Green et al., 2010). The left IFG and ATL are involved in the process of conceptual expansion (Abraham et al., 2012). Consequently, we define the creative-on/off as the balance between the DMN and the IFG network. When the former predominates over the latter, we enter into a creative-on state; otherwise, the cognitive system works in a routine creative-off state. Distinguishing these two states is interesting since it allows understanding the cognitive and behavioral differences we observe in an individual facing the same situation in two different moments. The creative-off state is a goal-oriented, routine-based, efficient system that generally produces good cognitive and/or behavioral outcomes. This state is governed by rules, such as the path-of-least-resistance rule, which allow the brain to achieve good solutions to typical problems in a short time. On the contrary, when an easy solution to a problem is not available, or when an individual is forced to go beyond the usual semantic or analogical associations, then the creative-off state cannot serve the purpose anymore and the usual and stable neurofunctional organization breaks down. The DMN takes the lead, and the short-range small-world organization leaves the field to long-range connections. In this way, the brain may expand its cognitive repertoire by linking together concepts, images, and experiences, thus allowing for more flexible thinking. It’s in this state that we may experience the semantic expansion that strings together concepts apparently distinguished to produce new ideas. Because the creativity-on state obviously requires more energy and a greater cognitive load, it is, therefore, less efficient than the creativity-off, which instead has been fine-tuned by evolution, education, and specific training. Naturally, the dynamic interaction between the DMN and the IFG network also involves many other neural networks. In particular, it is interesting to note that the attention network may be modulated by the DMN activity. When within the DMN the connection strength between mPFC and the PCC/prec is high, then the attention disengages from the external environment, thus shifting toward inner processes. This may favor mind-wandering as well as imagery activity. Instead, when a focus on external stimuli must be kept high, then pIPL-PCC/prec become predominant, thus inhibiting mind-wandering and other unrelated activities. In addition, the IFG network may modulate the attention focus when a complex task requires both concentration and the inhibition of distracting stimuli at the same time. The creative-on state is roughly similar to how the infant’s brain works. Indeed, Gardner (1982) defined the preschooler phase (between 3 and 6-year-old) as the Golden age of creativity, since infants show spontaneous and vibrant creativity in this period. This may be linked to the immaturity of pre-frontal cortices (Miller et al., 2012). Of course, creativity is different in childhood and adulthood. Adults’ creativity involves a number of different psychological mechanisms and is generally aimed to generate a well-defined product (Charles and Runco, 2001). That’s why the creativity-on state does not imply a generalized deactivation of prefrontal cortices, but a partial adjustment that permits both divergent and convergent thinking.

Considering the role of tDCS in the framework of creative-on/off, it is also interesting to appreciate how neurostimulation might be useful for promoting creativity outside the lab. In fact, we have seen that different tasks require different cortical targets, meaning that the multifaceted nature of creativity cannot be approached by a single tDCS program. For example, we could target the PFC to promote a more flexible cognitive process, and the ATL to promote verbal creativity. However, in real-life applications (e.g., to improve a manager’s creative decision making at the workplace) it’s difficult to define which creative mechanisms are the most important and which tDCS target will lead to better outcomes. These parameters are modulated by the individual’s characteristics, by contextual factors, and by the actual requirements of a given task. Furthermore, some creativity-related mechanisms are served by brain areas that are not possible to directly stimulate by non-invasive techniques. We argue that tDCS might be more useful in promoting a more general creative-on state, which will allow a spontaneous reorganization of the whole cognitive system. At this aim, it’s plausible to suggest that the IFG system will play a vital role in future programs that are intended to promote a creative-on state through tDCS, while other areas may be useful for specific purposes. Indeed, as revealed in a study by Meinzer et al. (2012) using fMRI after tDCS, the stimulation of the left IFG produced effects on a large network, including the anterior insula (salience network), the anterior temporal areas (language network) and the medial prefrontal cortex (DMN). Finally, it is noteworthy that the neurostimulation effect heavily depends on the cognitive task used. Thus, it will be important to test which task is able to elicit creative thoughts during which tDCS protocol.

In conclusion, our proposal is that starting by the creativity-on/off dynamic is quite easy to understand how tDCS or even other neurostimulation techniques may favor creativity. Indeed, since the creativity-on state may be considered a prodromal state, a kind of neural habitat suitable for creative ideas to survive, the role of tDCS is quite unspecific, modulating only the likelihood of a more flexible and imaginative thinking to arise. A modulation that might be useful in several different contexts. So, what kind of creativity is possible to stimulate? We argue that, following the classification by Kaufman and Beghetto (2009), promoting the elicitation of a creativity-on state by the inhibition of the IFG system and using proper cognitive tasks is possible to promote all form of creativity, from “mini creativity” (typical of infants) to everyday life “little creativity,” from “Pro-creativity,” (the one request in professional contexts) to “Big Creativity” (linked to object and important achievements). Of course, the actual results will depend also on contextual and personal characteristics not influenced by neurostimulation. Thus, it would be important for future research to address the topic of creativity within a more comprehensive framework.

## Author Contributions

The work was equally distributed between authors, each providing a substantial and intellectual contribution to the work, and approved it for publication.

## Conflict of Interest Statement

The authors declare that the research was conducted in the absence of any commercial or financial relationships that could be construed as a potential conflict of interest.
